# Supplementary material for: Mesenchymal Stem Cells and Formyl Peptide Receptor 2 Activity in Hyperoxia-Induced Lung Injury in Newborn Mice
Source: Int J Mol Sci. 2022 Sep 13;23(18):10604. doi: 10.3390/ijms231810604 (PMC9500980; doi:10.3390/ijms231810604)
Supplement: Supplementary file 1 [file ijms-23-10604-s001.zip › ijms-1834699-supplementary.pdf]

Supplementary Materials

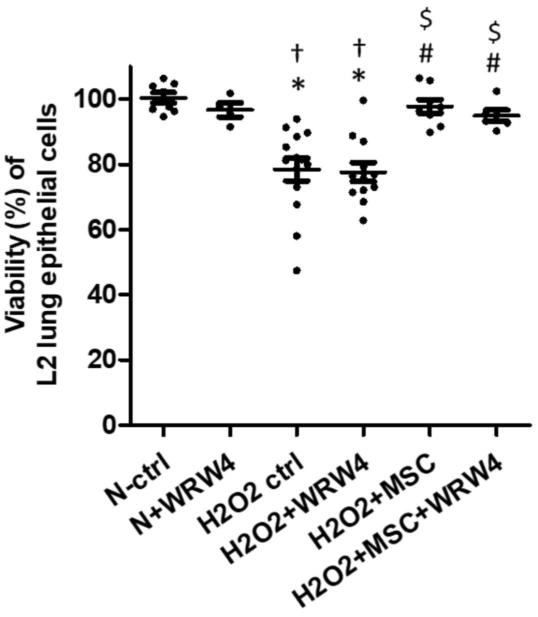

Supplementary Figure S1. Viability of L2 lung epithelial cells was measured after H_2O_2 (100μM) induction, with or without WRW4 (10μM) and MSCs (co-cultured in ratios of 5:1) treatment. Data are given as mean ± SEM. * P <0.05 vs. normal control group (N-ctrl). † P <0.05 vs. normoxia with WRW4-treated group (N+WRW4). # P < 0.05 vs. H_2O_2-treated control group (H_2O_2 ctrl). $ P < 0.05 vs. H_2O_2 with WRW4-treated control group (H_2O_2+WRW4)

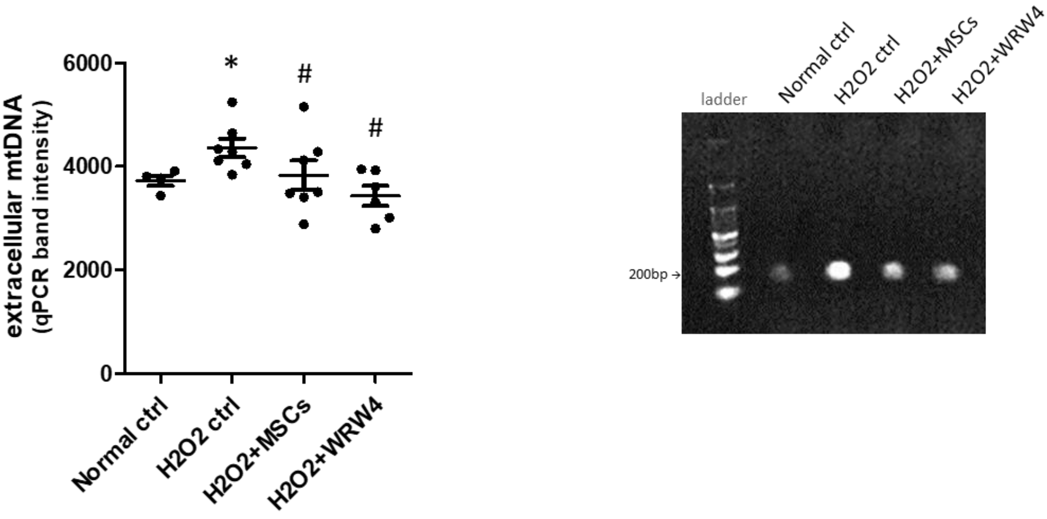

Supplementary Figure S2. Densitometric analysis of mitochondrial DNA (mtDNA) level in cell-free culture media of RAW264.7 alveolar macrophages. Data are given as mean ± SEM. * $P < 0.05$ vs. normal control group. # $P < 0.05$ vs. H_2O_2-treated control group (H2O2 ctrl).

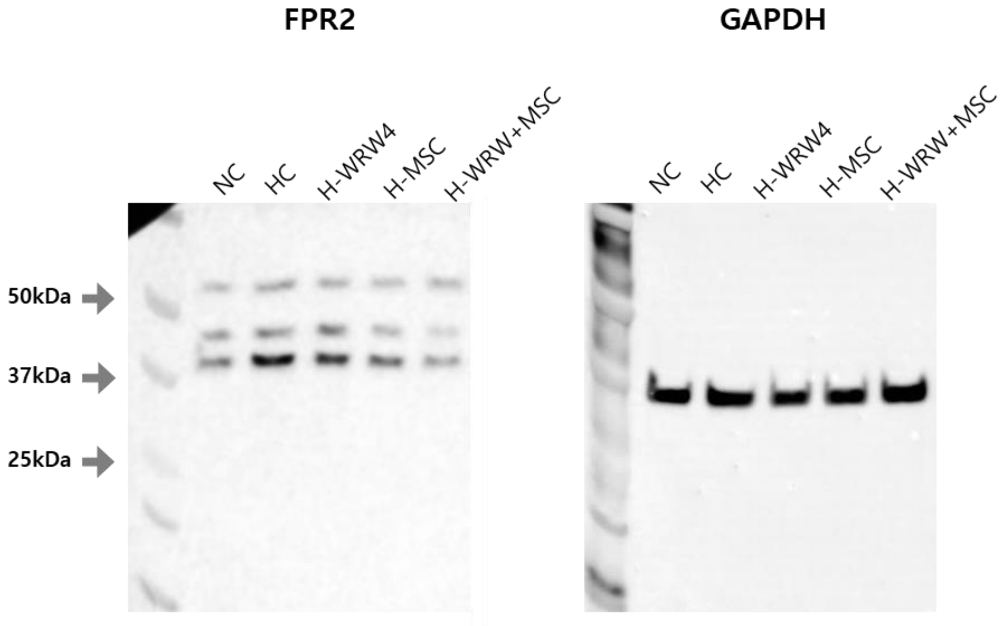

Supplementary Figure S3. Full-length Western blots of FPR2 (39 kDa) and glyxeraldehyde 3-phosphate dehydrogenase GAPDH (37 kDa), shown in Figure 1A.

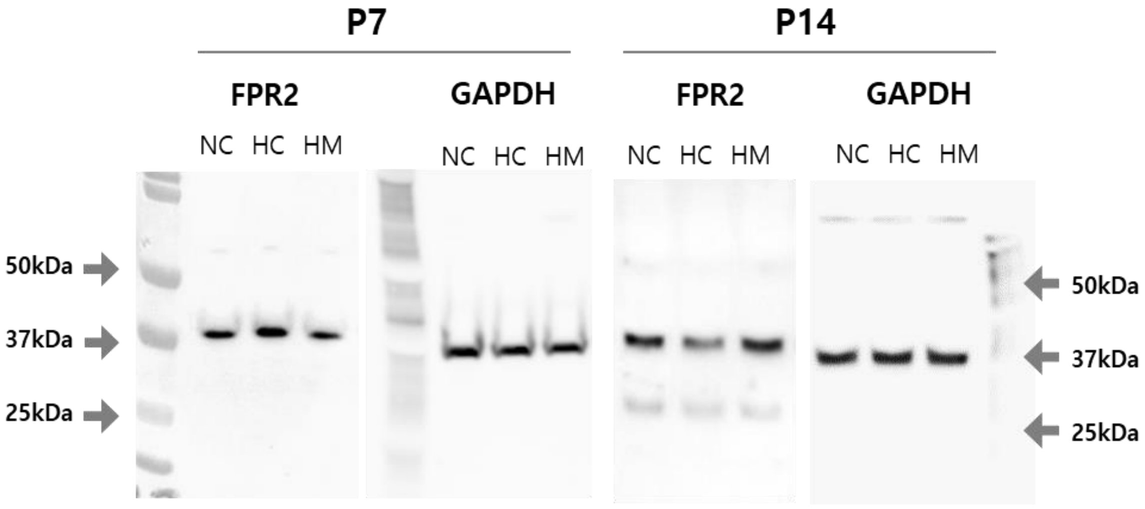

Supplementary Figure S4. Full-length Western blots of FPR2 (39 kDa) and glyxeraldehyde 3-phosphate dehydrogenase GAPDH (37 kDa), shown in Figure 2B.

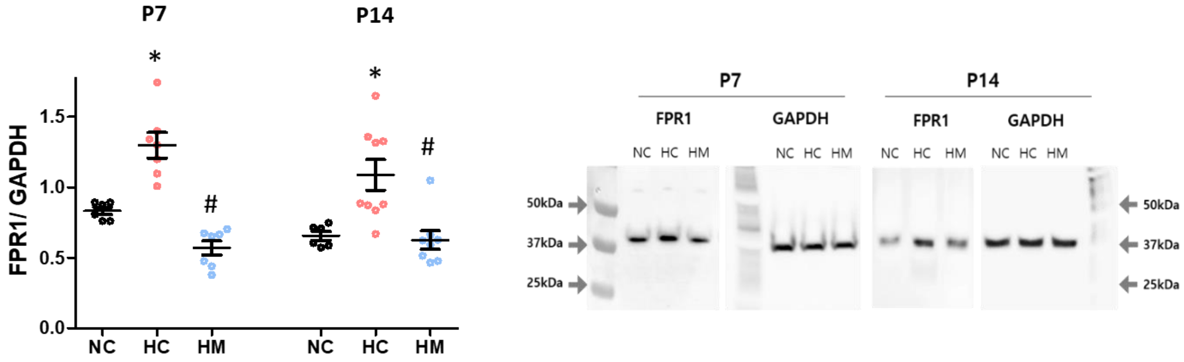

Supplementary Figure S5. Levels of FPR1 in wild-type mice lung at P7 and P14. Protein levels of FPR1, normalized to glyxeraldehyde 3-phosphate dehydrogenase (GAPDH), and representative western blots of FPR1 (39 kDa) and GAPDH (37 kDa). Data are given as mean ± SEM. *P < 0.05 vs. NC. #P < 0.05 vs. HC. NC, normoxia control; HC, hyperoxia control; HM, hyperoxia with intratracheal transplantation of human umbilical cord blood-derived MSC transplantation.

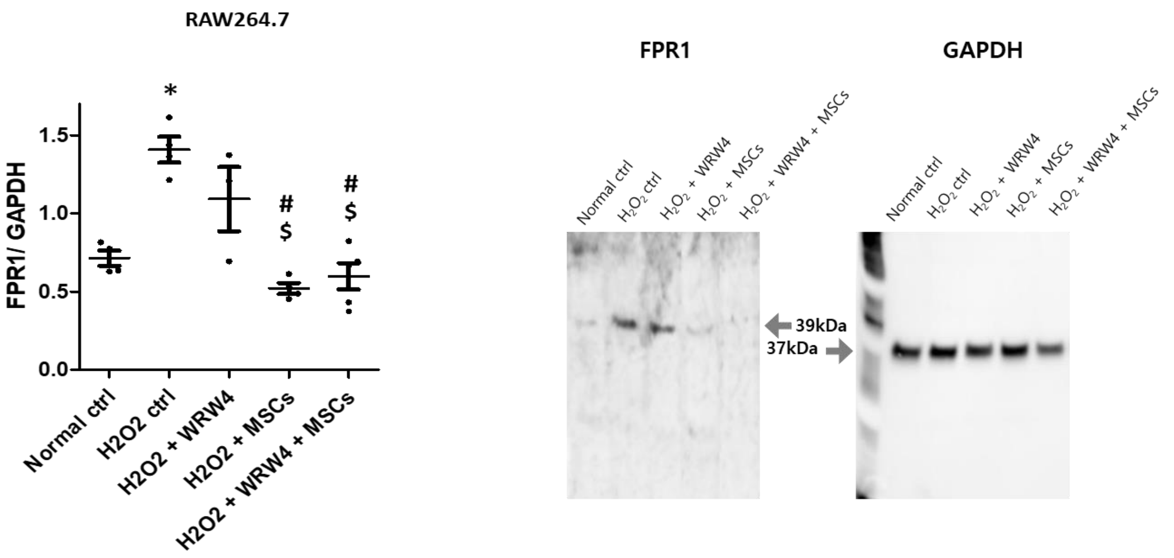

Supplementary Figure S6. Level of FPR1 in RAW264.7 alveolar macrophages. Protein level of FPR1, normalized to glyxeraldehyde 3-phosphate dehydrogenase (GAPDH), was measured after H_2O_2 (100 μM) induction, with or without WRW4 (10μM) and MSCs (co-cultured in ratios of 5:1) treatment, and representative western blots of FPR1 (39 kDa) and GAPDH (37 kDa). Data are given as mean ± SEM. * P <0.05 vs. normal control group. # P < 0.05 vs. H_2O_2-treated control group (H_2O_2 ctrl). $ P < 0.05 vs. H_2O_2 with WRW4-treated control group (H_2O_2+WRW4)

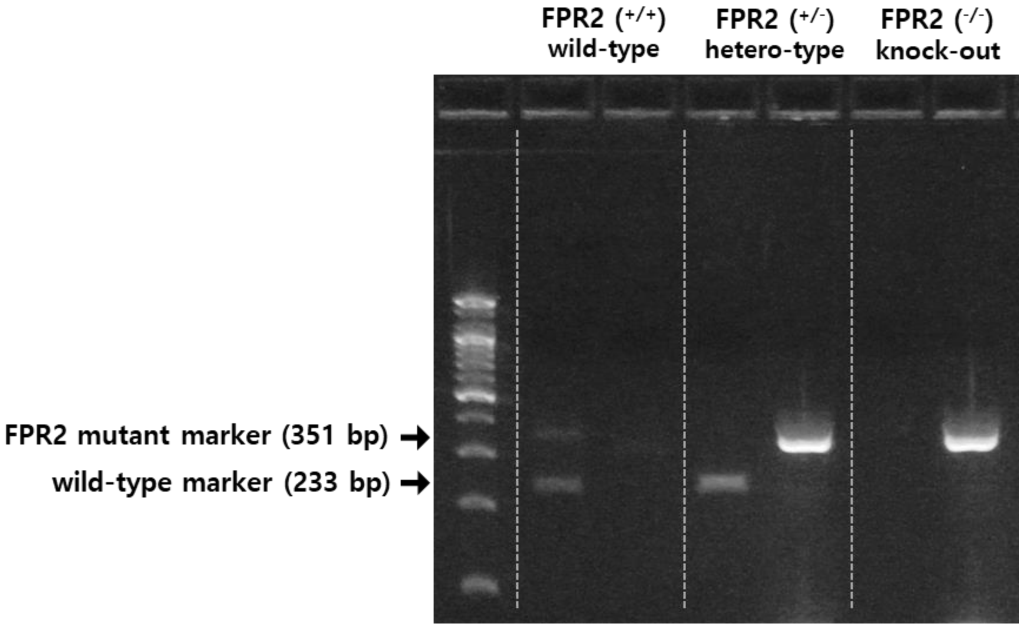

Supplementary Figure S7. Genotyping of FPR2 functional knockout (mutant) mouse. Mutated mRNA of FPR2 (351bp band) is distinguished from the wild type mRNA (233bp band) by PCR.
